# The Role of Circulating Tumor Cells in Chemoresistant Metastatic Breast Cancer

**DOI:** 10.3390/jcm10040684

**Published:** 2021-02-10

**Authors:** Lorena Alexandra Lisencu, Eduard-Alexandru Bonci, Alexandru Irimie, Ovidiu Balacescu, Cosmin Lisencu

**Affiliations:** 1 11th Department of Oncological Surgery and Gynecological Oncology, “Iuliu Hațieganu” University of Medicine and Pharmacy, 400012 Cluj-Napoca, Romania; lisencu.lorena@gmail.com (L.A.L.); airimie@umfcluj.ro (A.I.); cosminlisencu@yahoo.com (C.L.); 2 Department of Surgical Oncology, “Prof. Dr. Ion Chiricuță” Institute of Oncology, 400015 Cluj-Napoca, Romania; 3 Department of Functional Genomics, Proteomics and Experimental Pathology, “Prof. Dr. Ion Chiricuță” Institute of Oncology, 400015 Cluj-Napoca, Romania; obalacescu@yahoo.com; 4 11th Department of Medical Oncology, “Iuliu Hațieganu”, University of Medicine and Pharmacy, 400012 Cluj-Napoca, Romania

**Keywords:** metastatic breast cancer, chemoresistance, circulating tumor cells, epithelial-to-mesenchymal transition, cancer stem cells

## Abstract

Breast cancer is the most frequent form of cancer among women and is one of the leading causes of death. Two routes of the metastatic process have been described: linear and parallel progression. A key factor is represented by circulating tumor cells (CTCs). CTCs detach from the primary tumor or develop from cancer stem cells (CSCs) that undergo epithelial-to-mesenchymal transition (EMT). CTCs migrate to the distant site where the reverse process occurs and a new tumor arises. One of the key problems of metastatic disease is chemoresistance, which leads to treatment failure and, eventually, death. The aim of this review is to present up-to-date data regarding the role of CTCs in chemoresistance in metastatic breast cancer (MBC) patients. A search in Cochrane Library and MEDLINE databases was performed. A total of 125 articles were identified. The results of the final 12 eligible studies revealed that CTCs having stem cell features and those with mesenchymal features are aggressive subtypes of cells that survive chemotherapy, being responsible for chemoresistance and thus for disease progression in MBC patients. The hemodynamic shear stress, alongside dynamic changes among CTCs during the disease, is also an important disease progression factor.

## 1. Introduction

Breast cancer is the most frequent form of cancer among women. It was responsible for 13.3% of all cancer cases in Europe in 2020, and it is one of the leading causes of death, representing 7.3% of all cancer deaths [1]. Breast cancer is a heterogenous disease having different clinical outcomes. The heterogenicity is both inter- and intra-tumoral. Intertumoral heterogenicity is due to differences between patients, whereas intra-tumoral refers to cells from a tumor and its microenvironment expressing different phenotypic features and different metastatic potential [2].

The primary disease often completely responds to treatment, with satisfactory results, but the high mortality of this condition is due to metastatic disease. Once the disease spreads to distant sites such as the lung, liver, and bone, therapeutic options become limited and the treatment response unsatisfying, making this metastatic disease one of the leading causes of death in oncologic patients [3]. Metastatic invasion is an ineffective but highly lethal process that follows multiple steps. First, tumor cells migrate into the blood flow or lymphatic system, where they have to overcome multiple defense mechanisms of the host. Afterward, the malignant cells extravasate to a distant site, where tumorigenesis with the development of metastasis is initiated. During this process, less than 1% of disseminated tumor cells survive [4]. A recent study in the field presented two different routes of the metastatic process: linear progression and parallel progression. Linear progression is a step-by-step process in which metastatic potential is achieved progressively. Parallel progression has been demonstrated so far in human epidermal growth factor receptor 2 (Her-2)-positive tumors and shows that dissemination of cancer cells occurs early on before the primary tumor becomes clinically evident (the first step of metastasis takes place at the same time the primary tumor develops) [5].

To achieve metastatic potential, a series of morphologic and biochemical changes occurs in tumor cells and the tumor microenvironment. One of the most important steps is epithelial-to-mesenchymal transition (EMT), a process in which tumor epithelial cells, which are immobile, lose their epithelial features and gain mesenchymal properties, including the ability to migrate, invade, and disseminate [6]. This is in accordance with linear progression [5].

The majority of cells in breast cancer are represented by bulk tumor cells, but another type of cell is also present: cancer stem cells (CSCs). CSCs have many abilities such as regenerating bulk cells, inducing the EMT, and the capacity of self-renewal. CSCs also have the ability to switch between epithelial and mesenchymal phenotypes, meaning they switch from being proliferative to invasive, and vice versa. This could be a possible explanation for the fact that CSCs are more resistant to chemotherapy, as conventional chemotherapy targets proliferative cells [6].

CSCs from the primary tumor undergo EMT, become circulating tumor cells (CTCs), which migrate through the blood flow to distant sites, and then, due to a reverse process of mesenchymal-to-epithelial transition, CTCs regain their epithelial features and start to form a new tumor. Because of all the changes mentioned above, CTCs found in peripheral blood can express different features, such as stem cell, epithelial, or mesenchymal features [6]. Circulating tumor cells result from either detaching from the primary tumor and moving via blood flow to distant sites where they will give rise to metastasis, or forming as a result of the CSC EMT [5]. CSCs have been identified between CTCs, showing their ability to spread to distant sites through the blood flow [6].

Although it was initially thought that large tumors give rise to metastasis, later studies demonstrated that dissemination of tumor cells can occur from the earliest stages of invasive cancer. Only a few of the cells that enter the blood flow survive in the circulation, but this small subpopulation of cells that resists therapy holds metastatic potential, being an aggressive subset of cells. In patients without detectable metastasis, 10% to 40% present detectable CTCs [7,8]. CTCs can be obtained from the peripheral blood of patients, being minimally invasive and therefore having a real potential advantage for prognosis and treatment monitoring, but their identification is complex because they are very rare cells, with 1 CTC in 10^9^ nucleated blood cells. Nonetheless, in the last years, technology development has facilitated the detection of these cells and our ability to characterize them [7].

One of the key problems in metastatic disease is chemoresistance, with more than 90% of metastatic cancer patients becoming resistant to chemotherapy, which leads to treatment failure and, eventually, death. There are two pathways involved in chemoresistance: intrinsic and acquired. The problem with acquired chemoresistance is that the tumors may become resistant not only to the drugs that were first given, but also to other drugs through cross-resistance, reducing the efficacy of adjuvant therapy [9]. A possible explanation for chemoresistance is intratumor heterogeneity, with CSCs playing an important role through their capacity to self-renew, making these cells an aggressive subpopulation responsible for tumor recurrence. The tumor microenvironment is also important; it consists of inflammatory, vascular, and mesenchymal cells, which are all involved in controlling the features of CSCs [10].

CTC identification is an important independent prognostic factor in different types of aggressive solid tumors such as breast carcinoma, colorectal carcinoma, lung carcinoma, melanoma, and head and neck squamous cell carcinoma [11]. In prostate carcinoma, CTC number is a negative prognostic factor in terms of overall survival (OS) and progression-free survival (PFS) in both localized and metastatic disease [11,12]. CTCs are correlated with worse PFS and OS and with liver metastasis in colorectal carcinoma [11]. Another tumor site in which the presence of CTCs is associated with a worse prognosis is lung carcinoma [11]. When it comes to head and neck squamous cell carcinoma, CTC-positive patients have a worse PFS than CTC-negative patients, but clarifications about the presence of human papillomavirus in these cases are needed [11]. In esophageal cancer, the prognostic role of CTCs has been well established, as it was presented in a recent meta-analysis [13].

The prognostic role of CTC identification and characterization in terms of OS and PFS in metastatic breast cancer (MBC) is well known [6,11,14,15,16], but their role in monitoring and guiding therapy remains less clear. As CTCs have received much attention lately, and due to advances in technology that have facilitated their identification and characterization, we present here recent data about the role of CTCs in chemoresistance in patients with MBC.

## 2. Materials and Methods

This work is a systematic review of studies presenting information about the role of circulating tumor cells in chemoresistance among patients with MBC. An initial search was performed in the MEDLINE database using PubMed, searching the following MeSH terms: “breast cancer,” “circulating tumor cells,” and “chemoresistance” in best match without any filters. A total of 33 articles were found. Another search was performed in the MEDLINE database using PubMed, with the following MeSH terms: “breast cancer,” “circulating tumor cells,” and “antineoplastic drug resistance,” resulting in 44 articles. An advanced search was carried out in Cochrane Library by looking for the terms “breast cancer or mammary cancer” and “circulating tumor cells” and “resistant to therapy or drug-resistant” in the title, abstract, or keywords; 48 articles were found. A total of 125 articles were found until October 2020.

First, articles were screened, and duplicates were eliminated. Afterward, we applied a set of inclusion and exclusion criteria as presented in Table 1.

**Figure 1:**
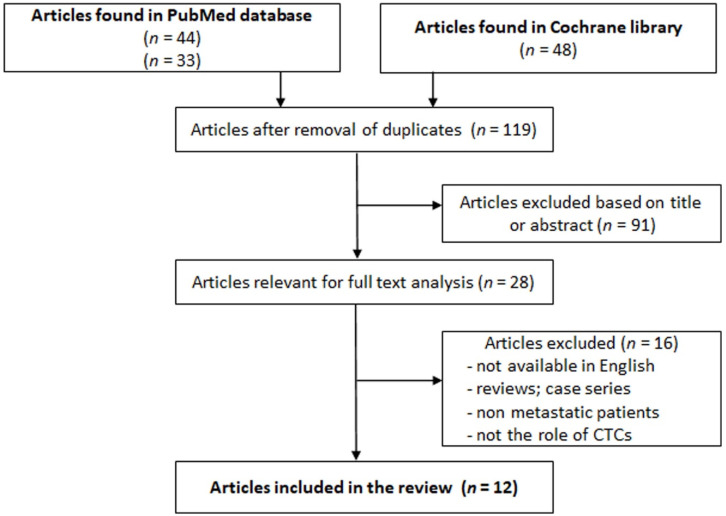
Study selection flow chart. CTCs: circulating tumor cells.

After a careful analysis of the 125 articles initially found, only 12 were suitable for inclusion (Figure 1; Table 2). Our study was conducted and reported using the PRISMA-P 2015 checklist [17].

## 3. Results

Of the 130 patients included in the study by Papadaki et al. [18], samples from 62 patients were available both before and after chemotherapy and were found to be CTC-positive at baseline. Triple immunofluorescence was performed using three main antibodies: cytokeratins (CKs) (markers for epithelial cells), aldehyde-dehydrogenase 1 (ALDH1)-anti-mouse (a marker for cancer stem cells), and TWIST 1-anti-rabbit (marker for epithelial-to-mesenchymal transition); four distinct subgroups of CTCs were obtained: non-CSCs epithelial-like (CTCs with epithelial markers but without features of stem cells), CSCs-positive epithelial-like (CTCs with both epithelial and stem cell markers), CSCs-positive partial EMT-positive (CTCs with stem cell features that are undergoing epithelial-to-mesenchymal transition), and non-CSCs partial EMT-positive (CTCs without stem cell features that are undergoing epithelial-to-mesenchymal transition). After chemotherapy, CTCs decreased or were eliminated in 61.3% of the patients, but a significant increase was observed among the CSCs-positive partial EMT subgroup, with this subset being detected in 30 patients after chemotherapy in comparison with 19 patients before chemotherapy. The number of CSCs-positive partial EMT CTCs per patient increased after chemotherapy. The CSCs-positive partial EMT CTCs were encountered only in Her2-negative patients and only among non-responders (stable or progressive disease after therapy), revealing that circulating tumor cells that possess both stemness and partial epithelial-to-mesenchymal markers are associated with a lack of response to therapy [18].

**Figure 2:**
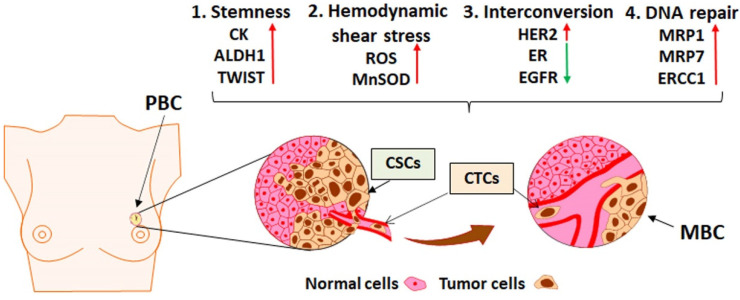
Chemoresistance of CTCs in metastatic breast cancer (MBC). The four mechanisms that underline CTC chemoresistance in MBC patients include (1) DNA Repair activation (upregulation of MRP1, MRP7 and ERCC1); (2) Stemness phenotype, characterized by activation of EMT factors (CK, ALDK1 and TWIST); (3) Hemodynamic shear stress, characterized by generation of ROS and expression of MnSOD; (4) Interconversion of Her2, ER, and EGFR expression in CTCs compared to the primary breast cancer (PBC).

Similar results were obtained by Gradilone et al. [19], who assessed the drug resistance profile and the correlation between primary tumors and metastasis in 42 MBC patients. The follow-up period was 2 years; blood draws were performed at baseline and every 9–12 weeks, while all the patients received chemotherapy according to the current guidelines. Tissue from the primary tumor was examined for estrogen receptors (ERs) and progesterone receptors (PRs), Ki-67, Her2 expression, and grading of the tumor differentiation. For CTC identification, the CELLection Dynabeads system was used. In the 28 patients that were found to be CTC-positive (≥5 CTCs/7.5 mL blood), the expression of multidrug resistance proteins (MRPs) was assessed. Of the 28 patients, 19 had a drug resistance profile based on the expression of the MRPs, and they had a worse prognosis than those who lacked MRP expression (PFS: 5.4 vs. 19.5 months). ALDH expression was also assessed in the CTCs as a stemness marker. Of 17 patients, 13 (76.47%) were found to be ALDH-positive; these patients were also drug-resistant. The results showed that a multidrug resistance profile can be identified by the expression of MRPs and the expression of ALDH, showing a more aggressive and resistant CTC population with stemness properties (Figure 2).

A circulatory system was created to analyze the impact of fluid shear stress on circulating tumor cells and the implication of this phenomenon in metastasis appearance. The experiments were performed on human breast cell lines. In all breast cancer lines, CTCs decreased as a result of being exposed to hemodynamic shear stress; circulating stem cells had much higher viability under shear stress. Ideally, cells responsible for metastasis survive the metastatic process, and they may be responsible for chemoresistance; therefore, cells that survived hemodynamic shear stress were then treated with chemotherapy (5-fluorouracil). Cells exposed to shear stress exhibited higher resistance to chemotherapy than those not exposed. After that, pharmacological inhibition of actomyosin was performed and this intervention enhanced chemotherapy resistance and upregulated the expression of genes responsible for multidrug resistance [20]. The effect of shear stress on circulating tumor cells was also studied by Fu et al. [21], who integrated a fluorescence resonance energy transfer (FRET) caspase sensor into an in vitro microfluidic system. The experiments were performed on breast cancer lines. The results showed that fluid shear stress enhanced the production of reactive oxygen species (ROS) as superoxide anions by the mitochondria, thereby initiating cancer cells apoptosis. One interesting observation was that the cancer cells expressing manganese superoxide dismutase (MnSOD) were more resistant to apoptosis induced by shear force, and they were more resistant to the chemotherapeutic agents, which exert their effect by generating ROS, for example, to doxorubicin. Higher levels of MnSOD were noted in metastatic cells compared to non-metastatic cells. MnSOD was found to be a protective mechanism against shear-stress-induced mitochondrial damage and against apoptosis. When MnSOD expression was analyzed among different breast cancer lines, its expression was heterogenous, and higher levels were expressed by MDA-MB-468 and MDA-MB-231, which are highly metastatic lines. When cells were exposed to shear stress in the microfluidic system for 18 h, 90% of the cells that survived were MnSOD-positive. This suggested the use of MnSOD as a marker for identifying CTCs with metastatic potential, which are predisposed to chemoresistance, and is a new potential therapeutic target (Figure 2).

Regarding changes to the circulating tumor cells, Xin et al. [20] developed a circulatory system comprising a silicone microtube, a peristaltic pump, and a syringe, which mimicked the tumor microenvironment. They reported the surviving circulating tumor cells exposed to hemodynamic shear stress changed their morphology, becoming elongated, suggesting cells that resist shear stress and show a higher resistance to chemotherapy may undergo epithelial-to-mesenchymal transition. This finding is supported by the observation that surviving cells expressed high levels of mesenchymal genes and lower levels of epithelial genes in comparison to the untreated cells [20].

The prognostic significance of dynamic CTC monitoring was assessed by Guan et al. [22] in a study performed on 108 Her2-negative MBC patients. Patients received chemotherapy with capecitabine and docetaxel for six or fewer cycles; after, patients that presented either stable disease or complete response were randomized and received capecitabine as maintenance therapy. For CTC identification, the CanPatrol filtration system was used, CTC phenotype was analyzed on the basis of EMT markers. Of the patients, 90 (83.3%) showed detectable CTCs at baseline. In a patient that received capecitabine, docetaxel, and afterward maintenance therapy with capecitabine in the first seven months, CTCs exhibiting a mesenchymal phenotype decreased; after seven months of maintenance therapy, the total CTC number decreased, but an increase in the number of mesenchymal CTCs was noted along with the appearance of distant metastasis. In another patient, increases in both the total number of CTCs and CTCs with mesenchymal features preceded evidence of disease progression on imaging within three months. This study concluded that both the total CTC number and mesenchymal CTC number have prognostic significance and are valuable for therapy monitoring.

Other changes among CTCs during the course of therapy were reported by Yu et al. [23] in a study in which CTC lines from six MBC patients with the estrogen-receptor-positive, luminal subtype, who presented progression under therapy or who did not receive any therapy, were cultured in vitro at different time points during the course of therapy. All patients included in the study received therapy according to the guidelines. CTC isolation was performed with CTC-Chip technology, which is a microfluidic system. In the CTC lines, mutations in two genes were observed that were not present in the primary tumor: PIK3CA and ER. Several drugs were tested on CTC lines as single agents or combined. As mutations in PIK3CA and ER genes appeared during the course of therapy, CTC lines harboring these mutations responded to different agents such as PIK3CA inhibitors and ER inhibitors, in comparison with the primary tumor. These results showed that cultures of CTCs from patients with advanced breast cancer can help with testing drug susceptibility and choosing the best therapy for each patient based on the pattern in which the cells change during therapy.

Dynamic changes in the CTCs were also found to be relevant by Jordan et al. [24] when CTCs from 19 ER-positive, Her2-negative MBC patients that received multiple courses of therapy were isolated using CTC-iChip technology. During the course of disease, 16 patients (84%) developed Her2-positive CTCs despite the primary tumor being Her2-negative. Her2 expression of the CTCs was reported to be bimodal: an increase in the Her2-positive subpopulation was concomitant with disease progression in patients with concomitant Her2-negative and Her2-positive CTC expression (Figure 2). Interconversion between Her2-negative and Her2-positive types was observed. To assess the importance of these changes, 55 drugs were tested, and a difference in the response of Her2-negative compared with Her2-positive CTCs was observed. These observations led to the conclusion that during the disease course and under therapy, in patients with advanced breast cancer, Her2-negative and Her2-positive CTCs coexist, and their ratio dynamically changes by interconversion. This could be one of the mechanisms involved in drug resistance. Similar results [25] were obtained from 36 MBC patients when changes that occurred among CTCs during six months of therapy were tracked. CTC identification and characterization were performed using both CellSearch and AdnaTest, adding antibodies against Her2 and EGFR (epidermal growth factor receptor) to recover CTCs that escaped from CellSearch identification. Blood draws were performed at five different time points during the course of first-line systemic therapy: at baseline and at one, three, four, and six months. Of the patients, 85% were considered CTC-positive at baseline using the agreement between the two isolation techniques. Gene expression was analyzed in 27 patients; it changed during the course of the disease: patients with HR-positive disease initially expressed ER, but after endocrine therapy or chemotherapy, the expression of ER was not detected anymore, whereas an increase in Her2 expression was noted in the CTCs during therapy. This was associated with disease progression and therapy resistance.

Similar outcomes were reported by Lavarov et al. [26] when 30 patients with early and advanced triple-negative breast cancer were subjected to CTC analysis before and after neoadjuvant chemotherapy using the breast select and detect system. CTCs were examined for the expression of mucin 1 (Muc-1) and Her2 oncoproteins. Five of 13 patients from the early breast cancer group and seven of 17 from the advanced breast cancer group had detectable CTCs before neoadjuvant therapy. After neoadjuvant therapy, CTCs were detected in only one patient with advanced breast cancer, and they did not have detectable CTCs at the moment of diagnosis; progression of the disease under chemotherapy was also noticed. In the aforementioned case, CTCs expressed Her2 even if the primary tumor was negative for ER, PR, and Her2, revealing that CTC Her2 expression is associated with therapy resistance and disease progression (Figure 2).

Regarding the tumor microenvironment, a comparison between the response of primary tumor cells (PTCs) and CTCs to chemotherapy was performed by Gong et al. [27]. Sixty patients were included in this study, of which 55 received chemotherapy according to the breast cancer guidelines. In vitro experiments were performed on tumor cells by adding epirubicin or cisplatin. For in vivo experiments, 6-week-old mice were injected with MDA-MB-231 breast cancer cells. After the tumor was detected and analyzed, chemotherapy (cisplatin at a dose of 5 mg/kg) was administered weekly for four cycles of therapy. CTCs and PTCs, obtained from five patients, were treated with epirubicin and cisplatin in vitro. PTC colonies were reduced by 87% with epirubicin and by 89% with cisplatin, whereas CTCs decreased by only 11.4% with epirubicin and 17.3% with cisplatin. These results suggested that CTCs are more resistant to chemotherapy than PTCs in vitro. The aforementioned results were then confirmed in vivo, when apoptotic CTCs and PTCs from 55 MBC patients were obtained before and after four cycles of chemotherapy. CTCs were found to be more resistant to chemotherapy. Another remark was the cells in suspension were more resistant to chemotherapy (epirubicin and cisplatin) than those in adherent cultures in eight samples. Many chemotherapeutic agents exert their effect by DNA damage, so any differences between the response to DNA-damaging drugs between PTCs and CTCs were assessed in five MBC patients. The authors found that chemotherapy-induced DNA damage was present in both PTCs and CTCs, but the DNA repair process was more efficient in CTCs than in PTCs. Detachment of CTCs from the matrix led to an increase in the levels of reactive oxygen species, a partial pre-activation of DNA damage checkpoints, and this was responsible for DNA damage and repair after chemotherapy (Figure 2). In vivo experiments were performed in mice by inoculating breast cancer cells, which were then treated with cisplatin and checkpoint inhibitors. The combination of cisplatin following checkpoint inhibitors resulted in an increase in apoptotic CTCs and suppressed liver and lung metastasis [27].

A total of 226 blood samples from 92 patients and 16 healthy controls were subjected to culture using laser-ablated microwells for eight weeks in a study by Khoo et al. [28]. Samples were analyzed at days 8 and 14; at day 8 the cells were in a monolayer, and at day 14 CTCs started to form proliferative clusters, and the other cells were slowly eliminated as cell debris. With the idea that CTCs can be used to evaluate the treatment response, 173 samples from 60 patients with breast cancer (early or metastatic) were analyzed pre- and post-treatment. It was found that CTC cluster formation decreased with the length of systemic treatment (88.6% pre-treatment vs. 42.6% after five weeks of treatment). These results suggested the use of cultured CTCs as a predictor for therapy response, with the hypothesis that the persistent CTC clusters could predict resistance to therapy.

Even though Twomey et al. [29] did not exactly analyze the role of CTCs in chemoresistance, we found their study relevant for our review because it examined the intrinsic characteristics of CTCs that may be responsible for their resistance to therapy. They developed in vitro CTCs in non-adherent conditions, as they usually are in the blood flow. They identified a mechanism involved in CTC escape from tumor necrosis factor (TNF) cytokine killing, which could contribute metastasis and chemoresistance. The expression of death receptors (DRs), which are correlated with the TNF death ligand, was tested; they reported DR5 expression was lower in the CTCs found in non-adherent conditions, which was linked to the delay in apoptotic response to the administration of human recombinant TNF-related apoptosis-inducing ligand, suggesting that DR5 could be a novel potential therapeutic target [29].

## 4. Discussion

On the basis of the analyzed studies we, found that both stem cell and mesenchymal features indicate the aggressive behavior of CTCs. Three [18,19,20] out of 15 studies determined that CTCs with stem cell features are an aggressive subset of cells, which are associated with a lack of response to chemotherapy. Three [18,20,22] studies revealed the selection of an aggressive subset of circulating tumor cells by EMT. As such, cells gain mesenchymal features, which are observed in circulating tumor cells resistant to chemotherapy. Related to prognosis, in one of the studies three features were found to be associated with a higher risk for relapse and with an increased risk of death: CTCs with CSCs and partial EMT features, triple-negative subtype, and liver metastasis in all cancer subtypes, except for the Her2-positive subtype [18].

Regarding the differences between primary tumor and circulating tumor cells, and the dynamic change in CTCs during the course of the disease, three studies [24,25,26] reported an increase in Her2-positive CTCs during therapy, even when the primary tumor was Her2-negative. The increase in the Her2-positive CTC population was associated with therapy resistance and disease progression. These observations suggested that dynamic identification and profiling of CTCs could help in determining therapy resistance over time, which would be useful for choosing the best therapeutic approach for each patient.

Genes associated with multidrug resistance were assessed in one [19] study, showing that those expressing multidrug resistance proteins had a shorter progression-free survival than those lacking MRP expression [19], and that characterizing CTCs according to their expression of multidrug resistance proteins is an important tool to predict drug resistance and therapeutic response. The drug resistance profile was also used in one case report [30]. The study by Gazzaniga et al. [30] analyzed expressions of MRPs in CTCs for a 64-year-old man diagnosed with hormone receptor (HR)-positive, Her2-negative MBC who received systemic treatment with epirubicin. It was found that MRP1 and MRP7, which are associated with resistance to anthracycline, were enhanced. This is consistent with the fact that the disease progressed after therapy with epirubicin. Hormone therapy was initiated with proof of stable disease at three months, but the disease progressed after the next three months. At the next blood examination, due to the lack of MRP5 expression (associated with resistance to carboplatin-gemcitabine) on the CTCs, a third-line therapy with a carboplatin-gemcitabine regimen was initiated. At the third count of CTCs under this regimen, no CTCs were detected. After four months, the stationary character of the disease was confirmed by computer tomography. This shows CTC monitoring and assessment of the drug resistance profile based on MRP expression are important tools to predict drug resistance and therapeutic response, providing valuable information earlier than imaging techniques.

In order to predict resistance to therapy, monitoring the presence of CTC clusters has been suggested, as CTC cluster formation was found to decrease with the length of systemic treatments, raising the hypothesis that persistence of CTC clusters could predict resistance to therapy [28].

Many chemotherapeutic agents damage DNA to achieve their therapeutic effect [27] [31]. The DNA repair process seems to be more efficient in CTCs than in primary tumor cells, according to Gong et al. [27]. When DNA checkpoint inhibitors were administered before chemotherapy, it was noticed that the DNA repair process decreased, suggesting a new potential therapeutic approach [27].

Chemoresistance mechanisms are complicated and not yet fully understood. There is a pressing need to understand the mechanisms involved in chemoresistance and to find new methods to overcome them. Better strategies for choosing the most appropriate treatment are needed [32].

Whether CTCs can be used as a tool to assess treatment response in breast cancer is a widely debated issue. Yan et al. [33] addressed this topic in a meta-analysis that included 50 studies and 6712 patients; they reported the CTC positivity rate decreased after therapy, suggesting CTCs could be helpful in monitoring treatment response in breast cancer patients. Another observation from this meta-analysis was that CTCs decreased after treatment in patients with different molecular subtypes of breast cancer, with the exception of the triple-negative subtype; this result agrees with the poor outcome of this subtype. Decrease in CTCs during the course of therapy is associated with a better prognosis in breast cancer patients, demonstrating that CTC identification can be a useful, non-invasive tool to evaluate the prognosis and response to therapy among breast cancer patients, and efforts to implement CTC monitoring in clinical practice are worthy.

## 5. Conclusions and Future Perspectives

Circulating tumor cells with stem cell features are an aggressive subtype responsible for chemoresistance and, thus, for disease progression in MBC patients. The aggressive behavior of CTCs is also gained by epithelial-to-mesenchymal transition, a process in which CTCs acquire partial EMT or mesenchymal features and are able to progress and invade.

CTCs dynamically change during chemotherapy, and this is one of the mechanisms involved in chemotherapy resistance. Some of the changes include accumulation of mutations in *PIK3CA* and *ER* genes, a more efficient DNA repair process, cluster formation, and an increase in Her2 expression. CTCs acquiring Her2 expression are more resistant to chemotherapy and are involved in disease progression.

In order to overcome CTC chemoresistance mechanisms, some solutions have been proposed. One of them is the use of cultured cells in order to examine CTC cluster development. If CTC clusters are present, a longer course of therapy could be needed as CTC clusters decrease with the length of systemic therapy.

There are cases in which primary tumor cells are Her2-negative, but along the course of chemotherapy an increase in Her2 expression of the CTCs is noticed, suggesting that Her2 expression could become a potential therapeutic target during the course of therapy.

Another difference between PTCs and CTCs is the DNA repair process. Even though chemotherapy-induced DNA damage is similar in PTCs and CTCs, the DNA repair process is more efficient in CTCs than in PTCs. In order to overcome this, administration of checkpoint inhibitors before chemotherapy has been proposed, as an increase in the number of apoptotic CTCs after their administration was noticed.

For the purpose of dividing MBC patients into groups according to the risk of disease progression, a multidrug resistance profile can be determined based on the expression of stemness markers and MRP expression. This is potentially beneficial when choosing chemotherapy agents on a susceptibility bases.

To summarize, the persistence of CTCs after chemotherapy is a negative prognostic marker associated with chemoresistance and disease progression. CTC monitoring, both in neoadjuvant and adjuvant settings, can help with choosing the best therapeutic strategy. Not only the number of CTCs but also their characteristics are important in monitoring the response to therapy, as some of their features, such as Her2 expression and stem cell and mesenchymal features, are associated with aggressive behavior and, therefore, with chemoresistance. There have been numerous attempts to overcome the complex mechanisms of chemoresistance.

Many studies in this review exposed some of the mechanisms implicated in chemoresistance and disease progression, but future studies are needed not only to fully characterize these mechanisms, but also to simplify the overall framework in order to implement effective treatments in clinical practice.

## Figures and Tables

**Table 1:** Inclusion and exclusion criteria for the analyzed studies.

Inclusion Criteria	Exclusion Criteria
Prospective and retrospective studies	Systematic reviews, case reports and meta-analyses
Studies available in English	Studies not available in English
Metastatic breast cancer	Non-metastatic breast cancer
Studying the role of CTCs * in chemoresistance	Other types of cancer apart from breast cancer

* CTCs, circulating tumor cells.

**Table 2:** Summary of studies investigating the role of CTCs ^i^ in chemoresistance in MBC ^iii^.

Author	Number of patients/Cell lines	Objectives	Results
Papadaki et al. [18]	130 patients	Prognostic significance of CTCs ^i^ with cancer stem cells and partial EMT ^ii^ properties Effect of first-line chemotherapy on these cells	CTCs expressing both stemness and partial EMT characteristics are associated with worse response to therapy
Gradilone et al. [19]	42 MBC ^iii^ patients	Assess if drug-resistant CTCs have predictive value and evaluate their stem-like properties Assess the expression of the MRPs ^iv^: MRP1: anthracycline and alkylants resistance, MRP5: cisplatin and carboplatin resistance, MRP7: taxanes resistance. MRP1 + MRP4: irinotecan, MRP5: 5 fluorouracil, MRP 1 + MRP7: vinca alkaloyds, MRP4 + MRP5: methotrexate resistance	Multidrug resistance profile can be identified by the expression of MRPs Expression of ALDH ^v^ helps create a more aggressive and resistant CTC population with stemness properties
Xin et al. [20]	Cell lines ^vi^: MDA-MB-231, MDA-MB-453, MDA-MB-468, MCF-7	Survival of tumor cells exposed to shear stress	Cells that survive shear stress possess some properties: partial EMT phenotype, CSC ^vii^ properties, low cell adhesion, and actomyosin play important roles in the survival of suspended tumor cells
Fu et al. [21]	Cell lines ^vi^: MCF-C3, MDA-MB-231, MDA-MB-468, T47D, SK-BR3, and 231-C3 In vivo: zebrafish circulation and mouse lung	Evaluate the apoptotic mechanism of CTCs both in vitro and in vivo	90% of the cells that survived shear stress were MnSOD ^viii^-positive, suggesting the use of MnSOD as a marker for identifying CTCs with metastatic potential
Guan et al. [22]	108 Her2 ^ix^-negative MBC patients	Assess the prognostic significance for dynamic CTCs identification	Total CTC number and mesenchymal CTC number have prognostic significance and are valuable for therapy monitoring
Yu et al. [23]	36 MBC patients; CTC cultures from six patients	Assess the drug responsiveness of the CTCs	Mutations in two genes (*PIK3CA* ^x^ and *ER* ^xi^) not present in the primary tumor were identified in CTCs Cultures of CTCs from patients with advanced breast cancer can help with testing drug susceptibility and choosing the best therapy
Jordan et al. [24]	19 ER-positive/Her2-negative MBC patients	Evaluate the expression of Her2 in the course of the disease in patients that were initially diagnosed with Her2-negative tumors	Ratio between Her2-negative and Her2-positive CTCs dynamically changes by interconversion, which could be one of the mechanisms involved in drug resistance
Aaltonen et al. [25]	36 MBC patients	Track the molecular changes that occur among CTCs during 6 months of therapy	ER expression decreased and Her2 expression increased during therapy An increase in Her2 expression of the CTC during therapy was associated with disease progression
Lavrov et al. [26]	13 early breast cancer patients and 17 advanced breast cancer patients	Evaluate the changes in the CTCs during neoadjuvant therapy	An increase in Her 2 expression was noticed in the CTCs after chemotherapy An increase in Her2 expression of the CTCs during therapy was associated with disease progression
Gong et al. [27]	60 patients 6 weeks old mice	Differences in the responses of PTCs ^xii^ to chemotherapy compared to CTCs	Both CTCs and PTCs respond to chemotherapy DNA induced damage, but the DNA repair process is more efficient in CTCs than in PTCs CTCs are more resistant to chemotherapy than PTCs
Khoo et al. [28]	92 breast cancer patients and 16 healthy subjects	Evaluate the use of CTC clusters in therapy monitoring	CTC clusters decrease with the length of systemic treatment
Twomey JD et al. [29]	Cell lines ^vi^: MDA-MB-231, MCF-7, and ZR75-1	Analyze the mechanisms involved in resistance of the CTCs	One of the mechanisms involved in chemoresistance is the downregulation of DR5 ^xiii^ expression

^i^ Circulating tumor cells; ^ii^ Epithelial-to-mesenchymal transition; ^iii^ Metastatic breast cancer; ^iv^ Multidrug resistance proteins; ^v^ Aldehyde-dehydrogenase 1; ^vi^ Breast cancer cell lines: MDA-MB-231, MDA-MB-453, MDA-MB-468, MCF-7; MCF-C3, T47D, SK-BR3, 231-C3 and ZR75-1; ^vii^ Cancer stem cells; ^viii^ Magnase superoxide dismutase; ^ix^ Human epidermal growth factor receptor 2; ^x^ Phosphatidylinositol-4,5-Bisphosphate 3-Kinase Catalytic Subunit Alpha; ^xi^ Estrogen receptor; ^xii^ Primary tumor cells; ^xiii^ Death receptor 5. MBC: metastatic breast cancer.

## Data Availability

Not applicable.
